# The long noncoding RNA DLGAP1‐AS2 facilitates cholangiocarcinoma progression via miR‐505 and *GALNT10*

**DOI:** 10.1002/2211-5463.13061

**Published:** 2020-12-31

**Authors:** Zhao Liu, Lili Pan, Xiaofang Yan, Xiuna Duan

**Affiliations:** ^1^ Department of Hepatobiliary and Pancreatic Surgery Jinan Central Hospital Cheeloo College of Medicine Shandong University Jinan China; ^2^ Department of Nuclear Medicine Central Hospital of Shan County Heze China

**Keywords:** cholangiocarcinoma, DLGAP1‐AS2, GALNT10, migration, miR‐505, viability

## Abstract

Cholangiocarcinoma (CCA) is a highly invasive malignant tumor with high mortality. Most cases of CCA are already advanced when they are detected, resulting in poor prognosis. As such, there is an ongoing need for the identification of effective biomarkers for CCA. The long noncoding RNA DLGAP1‐AS2 has been reported to have prognostic value in glioma and Wilms' tumor. Here, we investigated the function of DLGAP1‐AS2 in CCA. The differential expression of DLGAP1‐AS2 in CCA tissues and normal tissues was first examined using data from the The Cancer Genome Atlas database and then in CCA cell lines by quantitative RT‐PCR (qRT‐PCR). The target gene was predicted by bioinformatics analysis, and the binding sites were confirmed using luciferase assay. DLGAP1‐AS2 is up‐regulated in CCA, and high DLGAP1‐AS2 expression promotes cell viability and is associated with poor prognosis. Notably, DLGAP1‐AS2 acts as a sponge to suppress miR‐505 expression, and miR‐505 reduces the expression of *N*‐acetylgalactosaminyltransferase 10 (GALNT10) in CCA cells. Biofunctional experiments revealed that a miR‐505 inhibitor almost completely removed the inhibitory effect of si‐DLGAP1‐AS2 on CCA cell malignant progression, whereas the malignant phenotype of cells cotransfected with si‐DLGAP1‐AS2 and si‐GALNT10 was significantly reduced as compared with the control. In summary, the DLGAP1‐AS2/miR‐505/GALNT10 axis may contribute to regulating the malignant progression of CCA and may have potential as a novel target for CCA therapy.

## Graphical abstract

The long noncoding RNA *DLGAP1‐AS2* facilitates the malignant progression of cholangiocarcinoma via miR‐505 and *GALNT10* and may have potential as a novel target for cholangiocarcinoma therapy.

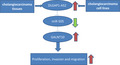

**Abbreviations**
CCA: cholangiocarcinoma
CCK‐8: Cell Counting Kit‐8
GALNT: *N*‐acetylgalactosaminyltransferase 10
lncRNA: long noncoding RNA
NC: negative control
qRT‐PCR: quantitative RT‐PCR
SD: standard deviation
TCGA: The Cancer Genome Atlas

Cholangiocarcinoma (CCA) is a highly invasive malignant tumor with high mortality, which originated from the epithelial cells of bile ducts [1]. In recent decades, despite the difference in incidence rate and etiology of CCA in the world, the incidence rate of CCA has been presented as an upward trend worldwide [2]. Until now, radical resection was the most valid treatment for CCA [3]. However, because of the insidious onset and rapid progress of CCA, most patients with CCA are in advanced stage when they are diagnosed clinically, thus losing the opportunity of radical operation, and have a poor prognosis [4, 5]. Hence it is important to master the molecular mechanism of CCA development for the study of effective biomarkers and therapeutic strategies.

Recent studies have found that long noncoding RNA (lncRNA) is a class of macromolecular noncoding RNA that mainly regulated gene expression in the form of RNA from three levels: transcription level, posttranscriptional regulation and epigenetics [6, 7, 8]. Importantly, lncRNA has been confirmed to act as a regulatory factor in tumor formation, invasion, metastasis and drug resistance mechanisms by affecting the expression of oncogenes [9, 10], including CCA. For example, *lncRNA‐EPIC1* is up‐regulated in CCA and accelerates the malignant biological behaviors of CCA by targeting *Myc* [11]. *lncRNA‐RMRP* contributes to accelerating the tumorigenesis and development of CCA by sponging miR‐217 [12]. Hence the development of lncRNA will provide the feasible targets for the targeted therapy of CCA. *lncRNA DLGAP1‐AS2* is located on human chromosome 18p11, and its role in the physiological process is rarely reported. Recently, two studies have confirmed its prognostic value in glioma [13] and Wilms' tumor [14]. Wherein, Miao *et al*. [13] indicated that *DLGAP1‐AS2* was highly expressed in gliomas and promoted the malignant phenotype of glioma cells by targeting *YAP1*. Nevertheless, the expression and function of *DLGAP1‐AS2* in CCA have not been studied.

Herein, we researched the level of *DLGAP1‐AS2* in CCA and assessed the impact of *DLGAP1‐AS2* on the malignant biological behaviors of CCA cells *in vitro*, as well as its potential molecular mechanism, which laid a theoretical foundation for the application of *DLGAP1‐AS2* as a therapeutic target of CCA.

## Materials and methods

### Data acquisition

We downloaded the RNA‐seq data from The Cancer Genome Atlas (TCGA) database. These data were applied to analyze the differential expression of *DLGAP1‐AS2*, miR‐505 and *N*‐acetylgalactosaminyltransferase 10 (*GALNT10*), which contains 36 patients with CCA and nine healthy control subjects. Moreover, the prognostic value of *DLGAP1‐AS2* was also assessed using data from TCGA database. Furthermore, patient information was extracted from TCGA database.

### Cell culture and transfection

CCA cell lines HUCCT1 and RBE were obtained from the Shanghai Cell Bank (Shanghai, China). Normal bile duct cell HEBEpics was purchased from the ScienCell Research Laboratories (Carlsbad, CA, USA). Dulbecco's modified Eagle's medium supplemented with 10% FBS and 100 U·mL^−1^ penicillin–streptomycin was used to culture the cells at 37 °C with 5% CO.

DLGAP1‐AS2 negative control (si‐NC, 5′‐TTCTCCGAACGTGTCACGT‐3′), si‐DLGAP1‐AS2#1 (5′‐CUGGCUGCGAAGUAAUAAAUU‐3′), si‐DLGAP1‐AS2#2 (5′‐GGCAGGAAAUUGUGUUUGU UU‐3′), pcDNA3.1‐DLGAP1‐AS2, miR‐505 inhibitor, miR‐505 mimic, negative control (miR‐NC) and si‐GALNT10 (5′‐ATGAGTACGCAGAGTACAT‐3′) were synthesized by Dharmacon. HUCCT1 and RBE cells were transiently transfected with these sequences by Lipofectamine 2000 as directed by the supplier. After 48 h, the transfection efficiency was measured using qRT‐PCR. The transfected cells were used for subsequent experiments.

### Total RNA extraction and qRT‐PCR

RNeasy Mini Kit (Qiagen, Dusseldorf, Germany) was used to isolate the total RNA from cells. One microgram of total RNA was used as a template, and cDNA was formed using the reverse transcription reaction system following the standard of the RT‐PCR kit. Then, qRT‐PCR was conducted to assess the mRNA levels of *DLGAP1‐AS2* and *GALNT10* with the SYBR Premix Ex Tap II kit (Takara, Dalian, China) following the supplier's instructions, and the data were normalized to GAPDH. The primer sequences of *DLGAP1‐AS2* and *GALNT10* were presented as follows: DLGAP1‐AS2: forward (F), 5′‐TTCCTGTCTTTCAGGATGAATGCC‐3′ and reverse (R), 5′‐TGGTAGCCTGTGGCGAGTTGAA‐3′; GALNT10: F, 5′‐GAGCCTTTGACTGGGAGATGTAC‐3′ and R, 5′‐GAGTTCCCAGAACCACTTCCGA‐3′; and GAPDH: F, 5′‐TAGATGACACCCGTCCCTGA‐3′ and R, 5′‐ACCTCCACCTGTCCTTAGTG‐3′.

### Western blotting

The cells were lysed with radioimmunoprecipitation assay buffer (containing protease inhibitor) to extract protein. Then, the proteins were separated with 10% SDS/PAGE gels and then transferred to polyvinylidene difluoride membranes. The membranes were sealed in 5% skimmed milk powder for 1 h, which then incubated with primary antibodies at 4 °C overnight. The primary antibodies were as follows: GALNT10 and GAPDH (1 : 1000; Abcam, Cambridge, MA, USA). After that, HRP‐conjugated secondary antibody was used to incubate the membranes for 2 h. Finally, Electro‐Chemiluminescence substrate was used to measure the blots.

### Bioinformatics prediction

LncBase (http://carolina.imis.athena‐innovation.gr/diana_tools/web/index.php?r=lncbasev2%2Findex) was applied to predict miRNAs regulated by *DLGAP1‐AS2*, and the target molecule of miR‐505 was predicted by starBase (http://starbase.sysu.edu.cn/).

### Luciferase assay

To confirm the results of bioinformatics prediction, we chose 293T cells with high transfection efficiency for luciferase activity detection. The *DLGAP1‐AS2* sequence was infixed with the pGL3 Basic vector. The luciferase reporter plasmid and miR‐505 inhibitor/NC were cotransfected into cells and cultured for 48 h. Then, the luciferase activity was detected using the Promega dual‐luciferase reporter gene detection kit following the supplier's standards. Also, according to the earlier steps, the luciferase reporter assay was conducted to assess the regulatory effect between *GALNT10* and miR‐505 as well.

### Proliferation assays

Cell Counting Kit‐8 (CCK‐8) and colony formation assays were carried out to assess the proliferative capacity of cells as described previously [15, 16]. In CCK‐8 assay, the cells were seeded into 96‐well plates (4 × 10^3^ cells per well) and cultured for 0, 24, 48 and 72 h. Then, CCK‐8 kit was used to detect the OD value at the specified time point as directed by supplier. Herein, 2 h before the detection, 10 μL CCK‐8 solution was appended to each well for culture.

For colony formation assay, in brief, about 400 cells were seeded into each medium and cultured at 37 °C with 5% CO_2_ for 7–14 days until the visible colonies appeared. Then, the cells were fixed with 4% paraformaldehyde and stained with crystal violet. Finally, we counted the number of colonies.

### Invasion and migration assays

Transwell assay was performed to appraise the ability of cell invasion and migration as mentioned earlier, with minor modifications [17]. The upper surface of the Transwell chambers was precoated with (invasion assay) or without (migration assay) Matrigel. Cells were inoculated to the upper chamber with the serum‐free medium, and the lower chamber was perfused with 500 μL complete medium. After 48 h, the remaining cells were removed from the upper surface, while the invaded or migrated cells on the lower surface were fixed with 4% paraformaldehyde and stained with crystal violet. Five visual fields were randomly selected to photograph and count under the microscope.

### Statistical analysis

spss 21.0 (IBM, New York, USA) statistical software was applied for the data analysis. All assays were performed three times, and the data were represented as mean ± standard deviation (SD) using the *t*‐test (two groups) and one‐way ANOVA (multiple groups) for comparison. The correlations between *DLGAP1‐AS2* and miR‐505, as well as *GALNT10* and *DLGAP1‐AS2*/miR‐505, were analyzed using Pearson’s correlation analysis. The survival curves were plotted using Kaplan–Meier survival analysis. A *P*‐value <0.05 represented that the difference was significant.

## Results

### Anomalous overexpression of lncRNA DLGAP1‐AS2 in CCA tissues and cell lines

**Fig. 1:**
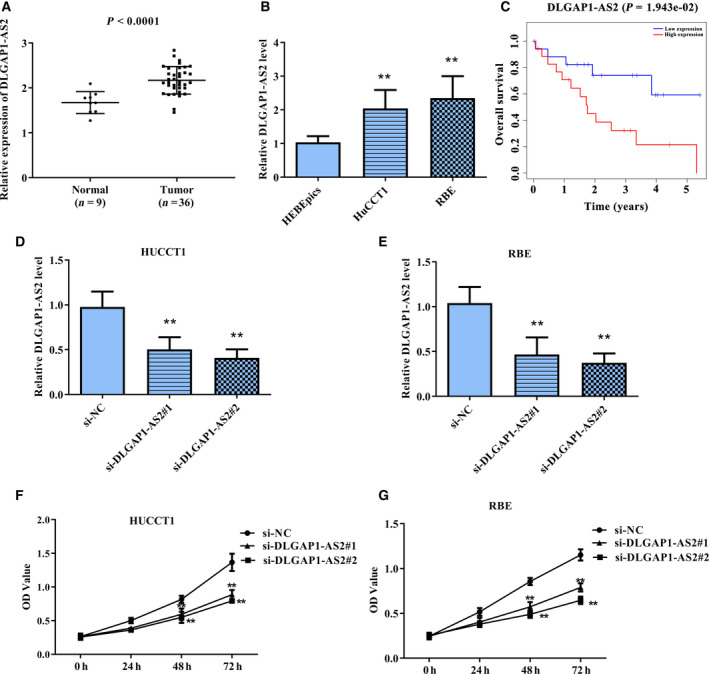
lncRNA DLGAP1‐AS2 was up‐regulated in CCA and contributes to promoting cell viability of CCA cells. (A) Bioinformatics analysis of DLGAP1‐AS2 expression in CCA tissues and normal tissues. *P* < 0.0001. (B) The levels of DLGAP1‐AS2 in CCA cell lines HUCCT1 and RBE, as well as normal HEBEpics cells. (C) Kaplan–Meier survival analysis of the relevance of DLGAP1‐AS2 expression and survival rate of patients with CCA. *P* = 0.0019. (D,E) The transfection efficiency of si‐DLGAP1‐AS2#1 and si‐DLGAP1‐AS2#2 in HUCCT1 and RBE cells was measured using qRT‐PCR. (F,G) The impacts of si‐DLGAP1‐AS2#1 and si‐DLGAP1‐AS2#2 on cell viability of HUCCT1 and RBE cells were detected using CCK‐8 assay. **P < 0.01. Error bar represents the mean ± SD derived from three independent experiments. Comparisons between groups were analyzed using *t‐*tests (two‐sided). OD, absorbance.

To detect whether *DLGAP1‐AS2* is abnormally expressed in CCA, we measured the level of *DLGAP1‐AS2* in CCA tissues and normal tissues based on the TCGA database. Patient information was displayed in Table S1. As presented in Fig. 1A, the *DLGAP1‐AS2* level was markedly enhanced in CCA tissues compared with that in normal tissues. Then, qRT‐PCR was conducted to confirm the *DLGAP1‐AS2* expression in CCA cell lines. Consistently, *DLGAP1‐AS2* presented the high level in CCA cell lines HUCCT1 and RBE compared with that in HEBEpics cells (Fig. 1B). Moreover, Kaplan–Meier survival analysis indicated that high *DLGAP1‐AS2* expression was related to poor outcomes of patients with CCA (Fig. 1C).

Considering the abnormal expression of DLGAP1‐AS2 in CCA and its prognostic value, we speculate that DLGAP1‐AS2 is involved in the malignant progression of CCA. First, we transfected si‐DLGAP1‐AS2#1 and si‐DLGAP1‐AS2#2 into HUCCT1 and RBE cells, respectively, to knock down DLGAP1‐AS2, and then qRT‐PCR was performed to detect the transfection efficiency. As presented in Fig. 1D,E, si‐DLGAP1‐AS2#1 and si‐DLGAP1‐AS2#2 both markedly decreased the DLGAP1‐AS2 level, and si‐DLGAP1‐AS2#2 had the higher efficiency. Then, CCK‐8 assay was performed to assess the cell viability. It can be seen from Fig. 1F,G that knockdown of DLGAP1‐AS2 led to a marked decline in the cell viability of both HUCCT1 and RBE cells. These findings revealed that *DLGAP1‐AS2* may contribute to promoting the malignant progression of CCA.

### lncRNA DLGAP1‐AS2 targeted miR‐505

**Fig. 2:**
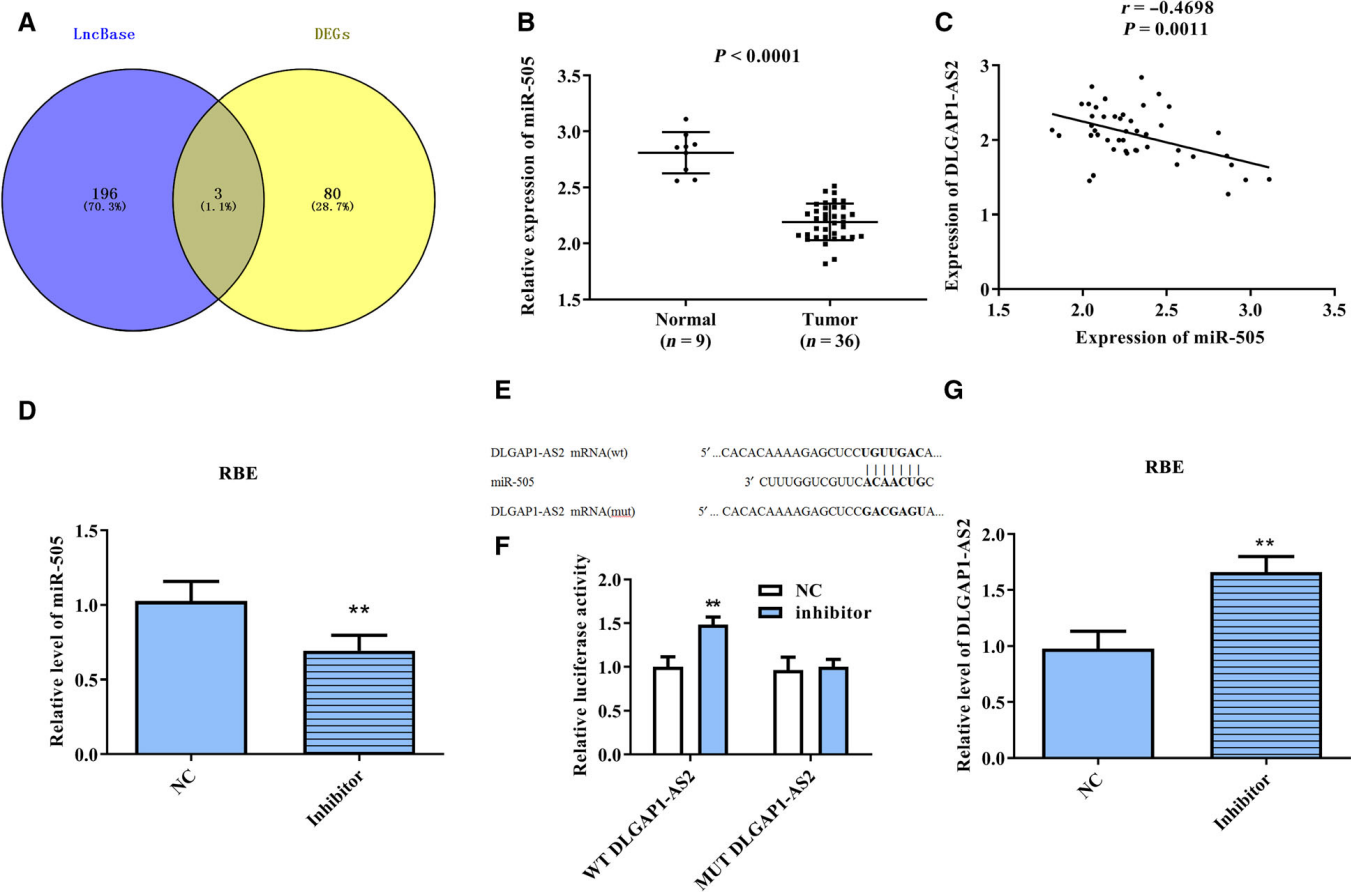
DLGAP1‐AS2 targeted miR‐505. (A) Wayne diagram was applied to ascertain the possible targets of DLGAP1‐AS2. (B) Bioinformatics analysis of miR‐505 expression in CCA tissues and normal tissues. *P* < 0.0001. (C) The correlation between miR‐505 and DLGAP1‐AS2. *R* = −0.4698; *P* = 0.0011. (D) The transfection efficiency of the miR‐505 inhibitor in RBE cells was measured using qRT‐PCR. (E) Comparison of miR‐505 with DLGAP1‐AS2 sequences. (F) Dual‐luciferase reporter assay was performed to confirm that miR‐505 directly binds to the 3′‐UTR of DLGAP1‐AS2. (G) The effect of the miR‐505 inhibitor on DLGAP1‐AS2 level was measured using qRT‐PCR. **P < 0.01. Error bar represents the mean ± SD derived from three independent experiments. Comparisons between groups were analyzed using *t‐*tests (two‐sided).

The potential targets of DLGAP1‐AS2 were predicted using miRDB and obtained 199 miRNAs. Then, the predicted miRNAs were intersected with the down‐regulated miRNAs analyzed in TCGA and obtained three common miRNAs (Fig. 2A), namely, hsa‐miR‐3163, hsa‐miR‐4705 and hsa‐miR‐505. Apparently, miR‐505 was lowly expressed in CCA tissues (Fig. 2B). Furthermore, Pearson’s correlation analysis indicated that the miR‐505 expression was negatively correlated with DLGAP1‐AS2 (Fig. 2C). In view of the earlier results, we selected miR‐505 for the follow‐up experiments. After transfection of the miR‐505 inhibitor, the level of miR‐505 decreased significantly, which indicated that the miR‐505 inhibitor actually inhibits miR‐505 (Fig. 2D). Based on the bioinformatics prediction, the binding site of DLGAP1‐AS2 and miR‐505 was presented in Fig. 2E. Subsequently, we conducted luciferase reporter assay to validate this prediction. As displayed in Fig. 2F, the luciferase activity of the WT‐DLGAP1‐AS2 reporter was markedly enhanced in the miR‐505 inhibitor group, while that of the MUT‐DLGAP1‐AS2 reporter with mutated binding site was almost unchanged in the miR‐505 inhibitor group. Moreover, we detected the effect of the miR‐505 inhibitor on DLGAP1‐AS2 level using qRT‐PCR. Interestingly, inhibition of miR‐505 led to a marked increase on DLGAP1‐AS2 level (Fig. 2G). These data indicated that *DLGAP1‐AS2* directly bound to miR‐505.

### *GALNT10*, a downstream molecule of miR‐505, was regulated via DLGAP1‐AS2

**Fig. 3:**
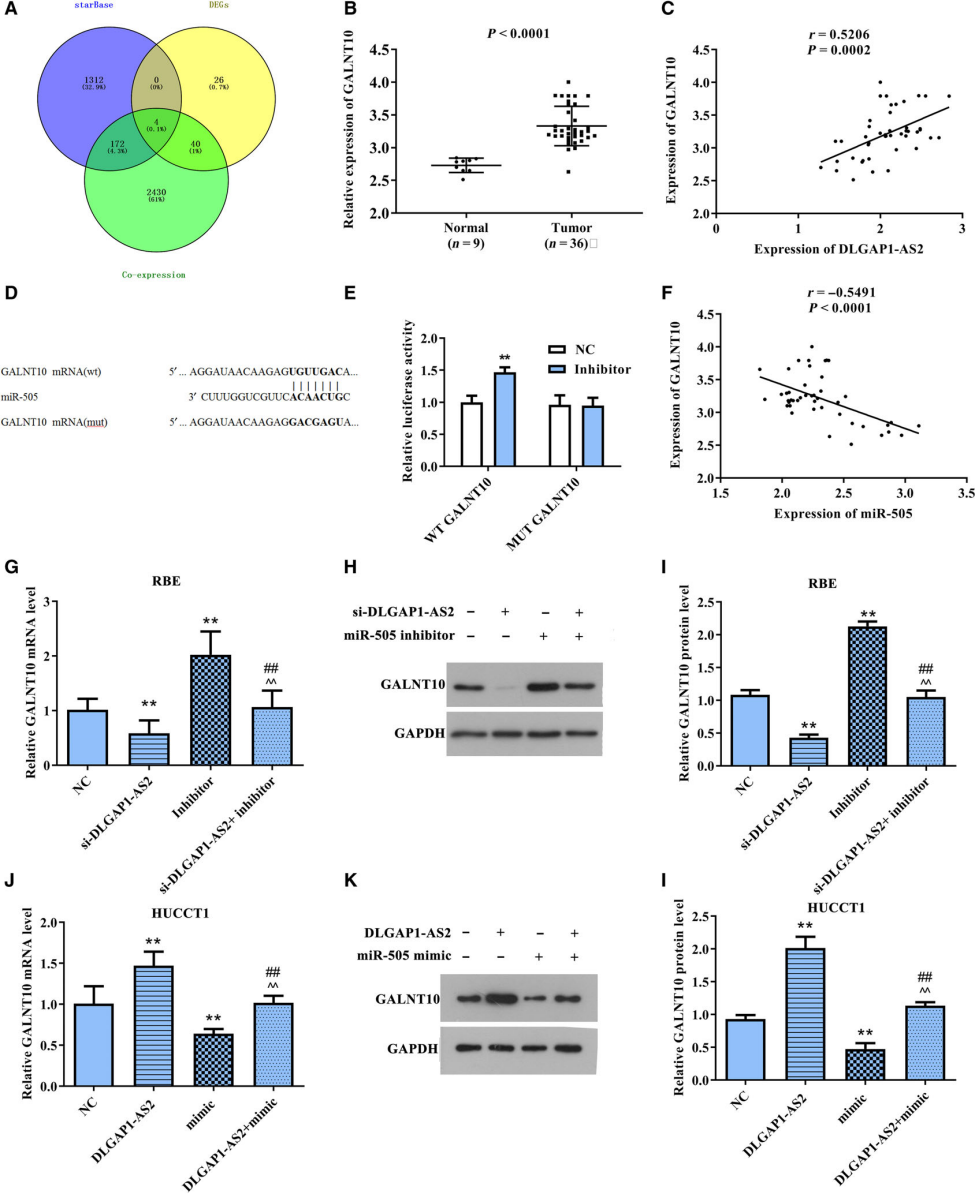
miR‐505 targeted GALNT10. (A) Wayne diagram was used to ascertain the possible targets of miR‐505. (B) Bioinformatics analysis of GALNT10 expression in CCA tissues and normal tissues. *P* < 0.0001. (C) The correlation between GALNT10 and DLGAP1‐AS2. *R* = 0.5206, *P* = 0.0002. (D) Comparison of miR‐505 with GALNT10 sequences. (E) Dual‐luciferase reporter assay was performed to confirm that miR‐505 directly binds to the 3′‐UTR of GALNT10. **P < 0.01. (F) The correlation between GALNT10 and DLGAP1‐AS2. *R* = 0.5206, *P* = 0.0002. (G–I) The impacts of si‐DLGAP1‐AS2, miR‐505 inhibitor and si‐DLGAP1‐AS2+miR‐505 inhibitor on GALNT10 mRNA and protein levels were detected using qRT‐PCR and western blotting. **P < 0.01 versus NC group, ^##^ *P* < 0.01 versus si‐DLGAP1‐AS2 group, ^^*P* < 0.01 versus miR‐505 inhibitor group. (J–L) The impacts of DLGAP1‐AS2, miR‐505 mimic and DLGAP1‐AS2+miR‐505 mimic on GALNT10 mRNA and protein levels were detected using qRT‐PCR and western blotting. **P < 0.01 versus NC group, ^##^ *P* < 0.01 versus DLGAP1‐AS2 group, ^^*P* < 0.01 versus miR‐505 mimic group. Error bar represents the mean ± SD derived from three independent experiments. Comparisons between groups were analyzed using *t‐*tests (two‐sided).

Next, starBase was used to predict the downstream molecules of miR‐505 and obtained 1488 target genes. Then, we analyzed the coexpression genes of DLGAP1‐AS2. Under the condition of *P* < 0.05 and correlation coefficient ≥ 0.3, 2646 genes with positive correlation were obtained. The predicted targets of miR‐505 and the coexpression genes of DLGAP1‐AS2 were intersected with the up‐regulated mRNA analyzed in TCGA database and obtained four common genes (Fig. 3A), namely, *ENTPD6*, *MECOM*, *GALNT10* and *HEY1*. The up‐regulated GALNT10 (Fig. 3B) with the largest difference and the strongest correlation (Fig. 3C) was selected for the study. The binding site of GALNT10 and miR‐505 was presented in Fig. 3D. Then, luciferase reporter assay revealed that the relative luciferase activity was markedly increased in the WT+miR‐505 inhibitor group compared with NC or mutant (MUT) groups (Fig. 3E). Our results verified that *GALNT10* is a downstream molecule of miR‐505.

Because Pearson’s correlation analysis also revealed that GALNT10 expression was negatively interrelated to miR‐505 expression and positively correlated with DLGAP1‐AS2 (Fig. 3F), we further measured levels of GALNT10 in the si‐DLGAP1‐AS2 group, miR‐505 inhibitor group and si‐DLGAP1‐AS2+miR‐505 inhibitor group, as well as in the DLGAP1‐AS2 group, miR‐505 mimic group and DLGAP1‐AS2+miR‐505 mimic. As presented in Fig. 3G–I, si‐DLGAP1‐AS2 restrained GALNT10 expression, whereas miR‐505 inhibitor up‐regulated its expression. Importantly, the inhibitory effect caused by down‐regulation of DLGAP1‐AS2 was attenuated after cells cotransfected with si‐DLGAP1‐AS2 and miR‐505 inhibitors. Contrarily, the GALNT10 expression was markedly increased in DLGAP1‐AS2 overexpressed cells but was obviously decreased in miR‐505 up‐regulated cells. Also, overexpression of miR‐505 can reverse the DLGAP1‐AS2‐induced promotion of GALNT10 levels (Fig. 3J–L). These outcomes revealed that GALNT10 levels were mainly mediated by the modulation of miR‐505 through *DLGAP1‐AS2*.

### DLGAP1‐AS2 promotes the malignant biological behaviors of CCA cells by increasing the GALNT10 level via restraining miR‐505

**Fig. 4:**
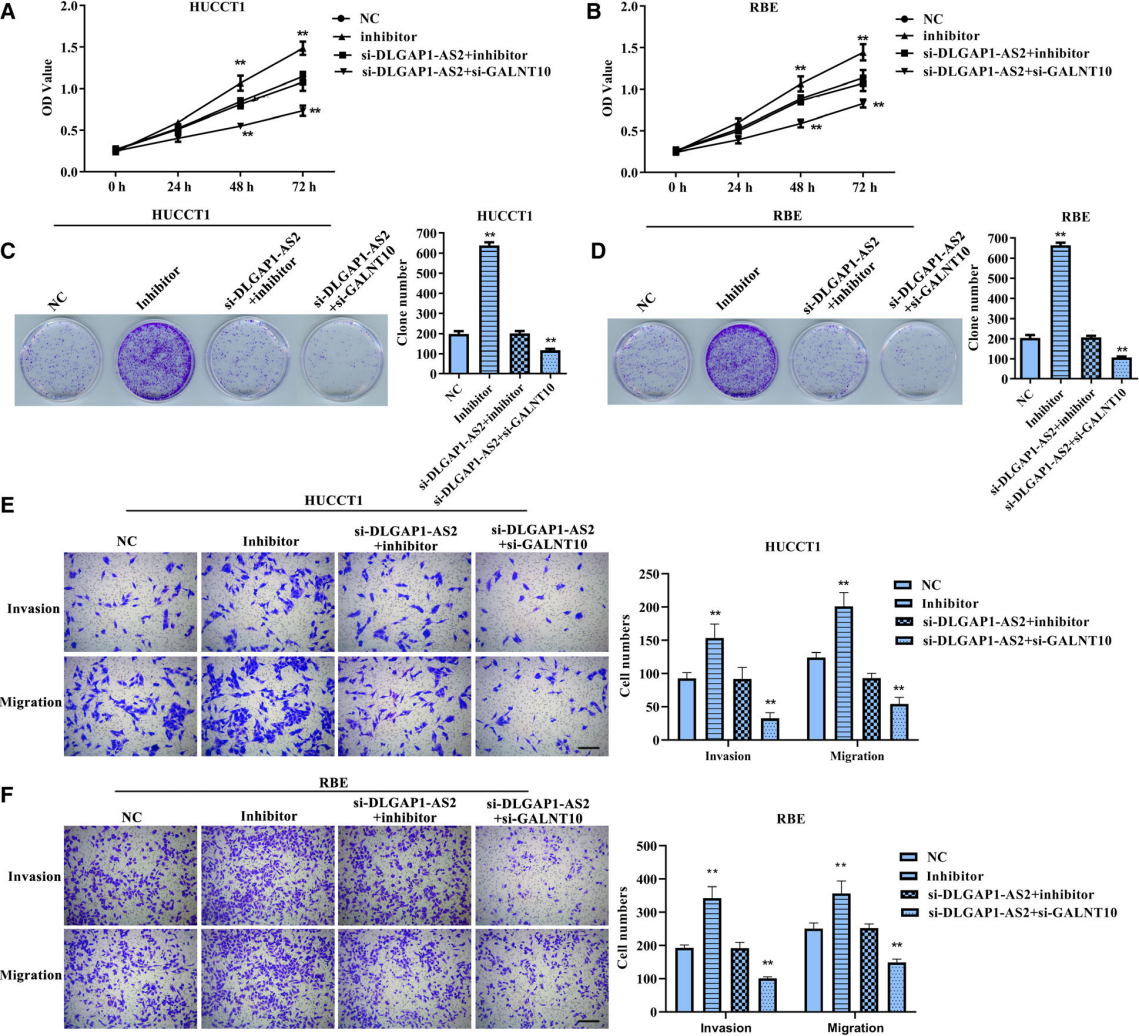
The impacts of miR‐505 inhibitor, si‐DLGAP1‐AS2+miR‐505 inhibitor and si‐DLGAP1‐AS2+si‐GALNT10 on malignant phenotype of HUCCT1 and RBE cells. (A,B) CCK‐8 assay for viability of HUCCT1 and RBE cells. **P < 0.01 versus NC group. (C,D) Colony formation assay for proliferation of HUCCT1 and RBE cells. **P < 0.01 versus NC group. (E,F) Transwell assay for migration and invasion of HUCCT1 and RBE cells. **P < 0.01 versus NC group. Scale bar: 200 µm. Error bar represents the mean ± SD derived from three independent experiments. Comparisons between groups were analyzed using *t‐*tests (two‐sided). OD, absorbance.

In view of the differential expression of DLGAP1‐AS2, miR‐505 and GALNT10, as well as their regulatory relationship, we conducted the functional experiments *in vitro* to verify the biological significance of the DLGAP1‐AS2/miR‐505/GALNT10 axis. CCK‐8 (Fig. 4A,B), colony formation (Fig. 4C,D) and Transwell assays (Fig. 4E,F) revealed that suppression of miR‐505 can markedly accelerate cell proliferation, migration and invasion compared with the NC group, whereas the phenotype of cells cotransfected with si‐DLGAP1‐AS2 and miR‐505 inhibitor was almost no different compared with the NC group. Also, after cells cotransfected with si‐DLGAP1‐AS2 and si‐GALNT10, the malignant biological behaviors of both HUCCT1 and RBE cells were significantly reduced compared with that of the NC group. These results demonstrated that *DLGAP1‐AS2* facilitates the malignant progression of CCA cells via regulating the miR‐505/GALNT10 cascade.

## Discussion

To our knowledge, most early‐stage CCAs are asymptomatic [1]. The lack of effective biomarkers leads to the loss of the best treatment opportunity for most patients. Herein, we expounded the possibility of the DLGAP1‐AS2/miR‐505/GALNT10 axis as a diagnostic marker and therapeutic target for CCA. As a result, high expression of DLGAP1‐AS2 in both CCA tissues and cell lines was associated with poor prognosis. Also, knockdown of DLGAP1‐AS2 decreased the viability of CCA cells. Importantly, miR‐505 was negatively regulated by DLGAP1‐AS2, and it inhibited GALNT10 expression. Then, functional experiments *in vitro* revealed that inhibition of miR‐505 could reverse the facilitating effects of si‐DLGAP1‐AS2 on cell phenotype, while the malignant biological behaviors of cells cotransfected with si‐DLGAP1‐AS2 and si‐GALNT10 were significantly inhibited compared with that of the NC group.

Extensive research reported that the abnormal expression of lncRNA in malignant tumors is closely related to the tumorigenesis, development and prognosis of cancer [18]. Some lncRNAs are highly expressed in tumors, which presented the characteristics of oncogenes [19, 20, 21]. As a little‐reported lncRNA, DLGAP1‐AS2 was up‐regulated in CCA tissues and cell lines, and down‐regulation of DLGAP1‐AS2 significantly decreased the cell viability of CCA cell lines HUCCT1 and RBE. These findings suggest that DLGAP1‐AS2 has tumor‐promoting properties.

To further explore the functional mechanism of DLGAP1‐AS2, we searched for its target and found that DLGAP1‐AS2 directly targets miR‐505. In recent years, multiple studies indicated that numerous microRNAs are aberrantly expressed in CCA and functioned as oncogenes or tumor suppressors in the proliferation, apoptosis, migration and invasion of CCA. For example, miR‐21 is highly expressed in CCA, which enhances the proliferation but restrains the apoptosis of CCA cells [22]. Contrarily, miR‐186 is down‐regulated in CCA, and overexpression of miR‐186 can suppress the proliferation of CCA cells [23]. Recently, miR‐505 has been widely studied in cancer. It is generally considered that miR‐505 has tumor‐suppressor properties, which has been confirmed to be down‐regulated in colorectal cancer [24], gastric cancer [25], endometrial cancer [26], osteosarcoma [27] and hepatocellular carcinoma [28]. Nevertheless, the role of miR‐505 in CCA has not been elucidated. In this work, we have reached a consistent conclusion. miR‐505 was down‐regulated in CCA tissues based on public data, and inhibition of miR‐505 led to a marked increase in cell proliferation, migration and invasion. Importantly, inhibition of miR‐505 can also reverse the si‐DLGAP1‐AS2‐induced inhibitory effect on cell viability. These data revealed that the DLGAP1‐AS2/miR‐505 axis contributes to regulating the malignant progression of CCA.

Interestingly, we found a negative correlation between miR‐505 and GALNT10 expression in CCA tissues. *GALNT10*, a member of the GALNT family, has been studied in a variety of cancers [29]. *GALNT10* can be applied as an independent prognostic factor for advanced ovarian serous carcinoma, which can predict poor prognosis and promote tumor progression [30]. Moreover, *GALNT10* is up‐regulated via HBV‐mediated inhibition of miR‐122 and functioned as an oncogene in the progression of hepatocellular carcinoma [31]. Our data indicated that GALNT10 was overexpressed in CCA tissues and related to the poor outcomes of patients with CCA. Notably, a biological function experiment revealed that knockdown of *GALNT10* and *DLGAP1‐AS2* together inhibited the proliferation, migration and invasion of tumor cells.

In conclusion, we elucidated the new function of lncRNA DLGAP1‐AS2 in facilitating the malignant progression of CCA cells by modulating the miR‐505/GALNT10 axis. These findings revealed the significance of the lncRNA DLGAP1‐AS2/miR‐505/GALNT10 axis in the malignant progression of CCA and supply a novel target for the CCA diagnosis and therapy.

## Conflict of interest

The authors declare no conflict of interest.

## Author contributions

ZL, LP and XD designed the study and collected data. ZL and XY analyzed the data. ZL and LP wrote the manuscript. ZL and XD reviewed and edited the manuscript. All authors read and approved the final manuscript.

## Supporting information


**Table S1.** Patient information.

## Data Availability

The data that support the findings of this study are available from the corresponding author upon reasonable request.
