# Supplementary material for: The long noncoding RNA DLGAP1‐AS2 facilitates cholangiocarcinoma progression via miR‐505 and GALNT10
Source: FEBS Open Bio. 2020 Dec 31;11(2):413–22. doi: 10.1002/2211-5463.13061 (PMC7876506; doi:10.1002/2211-5463.13061)
Supplement: Supplementary file 1 — Table S1. Patient information. [file FEB4-11-413-s001.docx]

| ID | Primary Diagnosis | Primary Site | Site of Resection of Biopsy | Normal Type |
| --- | --- | --- | --- | --- |
| TCGA-W5-AA34-11A-11R-A41I-07 | Cholangiocarcinoma | Liver and intrahepatic bile ducts | Intrahepatic bile duct | Solid Tissue Normal |
| TCGA-W5-AA31-11A-11R-A41I-07 | Cholangiocarcinoma | Liver and intrahepatic bile ducts | Intrahepatic bile duct | Solid Tissue Normal |
| TCGA-W5-AA2U-11A-11R-A41I-07 | Cholangiocarcinoma | Liver and intrahepatic bile ducts | Intrahepatic bile duct | Solid Tissue Normal |
| TCGA-W5-AA2R-11A-11R-A41I-07 | Cholangiocarcinoma | Liver and intrahepatic bile ducts | Intrahepatic bile duct | Solid Tissue Normal |
| TCGA-W5-AA2X-11A-11R-A41I-07 | Cholangiocarcinoma | Other and unspecified parts of biliary tract | Extrahepatic bile duct | Solid Tissue Normal |
| TCGA-W5-AA2I-11A-11R-A41I-07 | Cholangiocarcinoma | Liver and intrahepatic bile ducts | Intrahepatic bile duct | Solid Tissue Normal |
| TCGA-W5-AA30-11A-11R-A41I-07 | Cholangiocarcinoma | Liver and intrahepatic bile ducts | Intrahepatic bile duct | Solid Tissue Normal |
| TCGA-W5-AA2Q-11A-11R-A41I-07 | Cholangiocarcinoma | Liver and intrahepatic bile ducts | Intrahepatic bile duct | Solid Tissue Normal |
| TCGA-ZU-A8S4-11A-11R-A41I-07 | Cholangiocarcinoma | Liver and intrahepatic bile ducts | Intrahepatic bile duct | Solid Tissue Normal |

**Supplementary Table.1 patient information**
